# Evaluation of Neurosurgery Consultations in Hospitalized Geriatric Patients During and After the COVID-19 Pandemic

**DOI:** 10.3390/medicina61020315

**Published:** 2025-02-11

**Authors:** Hakan Kina, Hakan Yavuzer

**Affiliations:** 1 Department of Neurosurgery, Istinye University Gaziosmanpasa Medical Park Hospital, 34250 Istanbul, Türkiye; 2 Department of Internal Medicine, Istinye University Gaziosmanpasa Medical Park Hospital, 34250 Istanbul, Türkiye

**Keywords:** geriatric neurosurgery, COVID-19, consultation patterns, geriatric patient care, neurosurgical outcomes

## Abstract

*Background and Objectives*: This study aims to evaluate neurosurgery consultations for elderly patients during and following the COVID-19 pandemic. *Materials and Methods*: This study included patients aged 65 and older who were hospitalized in non-neurosurgery departments at Istinye University Gaziosmanpasa Medical Park Hospital and were referred for neurosurgery consultations between 1 April 2020 and 31 May 2024. Patients in the intensive care unit and emergency department were excluded. The period from 1 April 2020 to 30 April 2022 was defined as the pandemic period, and from 1 May 2022 to 31 May 2024 as the post-pandemic period. *Results*: A total of 123 patients were included in this study, with 57 from the pandemic period and 66 from the post-pandemic period. The average age during the pandemic period was 73.45 years (range: 65–93), compared to 71.09 years (range: 65–94) in the post-pandemic period. During the pandemic, 26.3% of patients were recommended for physical therapy and rehabilitation, 24.6% were advised to undergo surgery, 19.3% received neurology consultations, and 17.5% received medical treatment. In the post-pandemic period, 37.9% were recommended for surgery, 16.7% for neurology, 13.6% for physical therapy and rehabilitation, and 7.6% for medical treatment. Overall, 56.4% of patients accepted surgery. *Conclusions*: Despite the high prevalence of comorbidities in geriatric patients, appropriate neurosurgical referrals significantly improve treatment success, enhance quality of life and mobility, and reduce mortality. We therefore recommend earlier and more attentive referrals to neurosurgery for elderly patients with relevant symptoms to facilitate timely and effective interventions.

## 1. Introduction

Improvements in living conditions and healthcare services have substantially contributed to a notable increase in life expectancy worldwide [1]. By the late 19th century, the likelihood of reaching the age of 65 was less than 50%, whereas, a century later, this figure had nearly doubled to approximately 90% [2]. As the global elderly population continues to grow, the demand for various surgical services, including neurosurgery, is expected to rise correspondingly [3,4]. Geriatric patients constitute a unique group, often requiring specialized attention due to a high prevalence of comorbidities and distinct physiological challenges, particularly during surgical interventions [5]. It is imperative to address the increasing demand for geriatric neurosurgical care and proactively prepare for the planning and development of healthcare services. Furthermore, it has been indicated that future research will not only enhance patient outcomes but also establish best practices and policies in the field of geriatric neurosurgery [6]. The Coronavirus Disease 2019 (COVID-19) pandemic was a major public health crisis that significantly altered the way patients sought and received care. Due to the highly contagious nature of COVID-19 and its association with high morbidity, many healthcare centers faced increased pressure on resources while managing an unprecedented volume of virus-related admissions [7]. The COVID-19 pandemic forced healthcare providers to refocus care efforts, prioritizing acute care for COVID-19 patients and those with severe medical conditions [8]. This had a profound impact on global public health, as indicated by predictive models showing that the death rates from non-communicable diseases could surpass the cumulative number of COVID-19 cases and related deaths, which were experiencing rapid, near-exponential increases [9,10]. The World Health Organization declared COVID-19 a pandemic in March 2020, followed by a nationwide lockdown in Turkey in April 2020, significantly reducing elective surgeries and outpatient services. Public health interventions, coupled with the subsequent city-wide lockdown, contributed to mitigating the virus’ impact, while simultaneously exerting diverse effects on the dynamics of urban life and the provision of healthcare services [11]. By the end of April 2022, life in Turkey largely returned to normal [12,13]. The COVID-19 pandemic represented a major public health crisis that notably impacted how geriatric patients accessed and received treatment [9,10]. Many specialties, including neurosurgery, have reported a reduction in the number of patients treated through outpatient visits, referrals, and both elective and emergency surgeries during the pandemic period, excluding COVID-19-related emergencies [7,8,9,10,14,15,16,17,18,19]. Additionally, studies in geriatric neurosurgery frequently focus on specific conditions, such as trauma, tumors, and aneurysms [20,21,22]. The geriatric population has been severely affected by the pandemic, with high mortality rates, and the secondary impacts of the COVID-19 pandemic on this group have not been fully elucidated [23]. While surgical interventions have primarily been directed toward patients with life-threatening conditions, the effects of the COVID-19 pandemic on neurosurgical procedures in geriatric individuals have only been partially assessed [7,8,14,15,16,17,24]. Furthermore, insights into the impact of the COVID-19 pandemic on specific areas of surgery in geriatric patients, as well as its influence on diagnostic procedures, remain insufficiently explored. This study aims to evaluate neurosurgery consultations for elderly patients during and after the COVID-19 pandemic.

## 2. Materials and Methods

This study included patients aged 65 and older who were hospitalized in non-neurosurgery departments at Istinye University Gaziosmanpasa Medical Park Hospital and were referred for neurosurgery consultations between 1 April 2020 and 31 May 2024. The period from 1 April 2020 to 30 April 2022 was defined as the pandemic period, and from 1 May 2022 to 31 May 2024 as the post-pandemic period. Patients in the intensive care unit, emergency department, and physical therapy and rehabilitation department were excluded. Patients with unavailable prior medical records or who were lost to follow-up after discharge were excluded. Data on patient demographics, comorbidities, previous surgical history, prior neurosurgery outpatient visits, and radiological findings were retrieved from the hospital’s health information system. Additional information collected retrospectively included the referring department, the reason for the neurosurgery consultation, the current hospitalization diagnosis, symptoms, surgical indications, types of surgical procedures, and treatment modalities. The average follow-up period was calculated from the date of consultation until the latest available record in the health information system as of September 2024. Surgery-related mortality was defined as occurring within 30 days from surgery [22,25,26,27]. Overall mortality data were confirmed through the health information system. The study was approved by the Istinye University Ethics Committee (No: 24-153). Statistical analyses were conducted using SPSS (Version 22.0, IBM Corp., Armonk, NY, USA). Data distribution normality was assessed using the Kolmogorov–Smirnov test. Descriptive statistics were presented as mean ± standard deviation (SD) or median (min–max), based on data distribution. Relationships among categorical variables were analyzed with the chi-square test, and Fisher’s exact test was applied for cases with expected frequencies below 5 to ensure accurate results. The Mann–Whitney U test was used for comparisons between two independent groups when normality assumptions were not met. A *p*-value of less than 0.05 was considered statistically significant for all tests.

## 3. Results

A total of 123 patients were included in this study, with 57 from the pandemic period and 66 from the post-pandemic period. The average age during the pandemic period was 73.45 years (range: 65–93), compared to 71.09 years (range: 65–94) in the post-pandemic period. Males constituted 40.4% of the patients in the pandemic period and 54.5% in the post-pandemic period. COVID-19 positivity was reported in 26.3% of patients during the pandemic and decreased to 10.6% in the post-pandemic period. The hospitalization rate for surgical intervention increased significantly, from 5.3% during the pandemic to 2 1.2% post-pandemic (*p* = 0.011). The rate of patients in whom medical treatment initiated by their primary physicians was deemed insufficient was 23.4%, with 87% of these cases being non-tumor lumbar spine pathologies. In the pandemic period, 17.5% of patients had a history of neurosurgical procedures, which significantly increased to 36.4% in the post-pandemic period (*p* = 0.02). Trauma history was present in 10.5% of patients during the pandemic, rising to 19.7% post-pandemic (Table 1).

**Figure 1:**
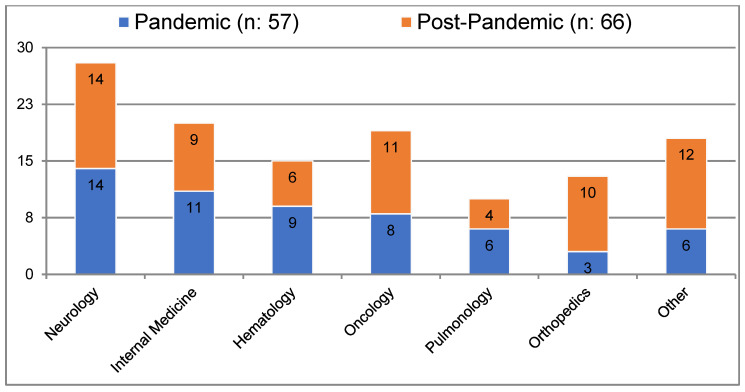
Patient hospitalization departments during consultation.

During the pandemic, most consultations were requested by the neurology department (24.6%), followed by internal medicine (19.3%), hematology (15.8%), oncology (14%), and pulmonology (10.5%). In the post-pandemic period, neurology again accounted for the majority of consultation requests (21.2%), followed by oncology (16.7%), orthopedics (15.2%), and internal medicine (13.6%) (Figure 1). In both periods, malignancy was the leading cause of hospitalization, with rates of 19.3% and 24.2%, respectively (Table 2).

The most frequent reason for consultation during the pandemic was back and leg pain (36.8%), which decreased significantly to 19.7% in the post-pandemic period. In contrast, motor weakness became the most common reason for consultation post-pandemic, at 25.8%. All patients with a history of prior surgery who required consultation were in the post-pandemic period (Table 3).

**Figure 2:**
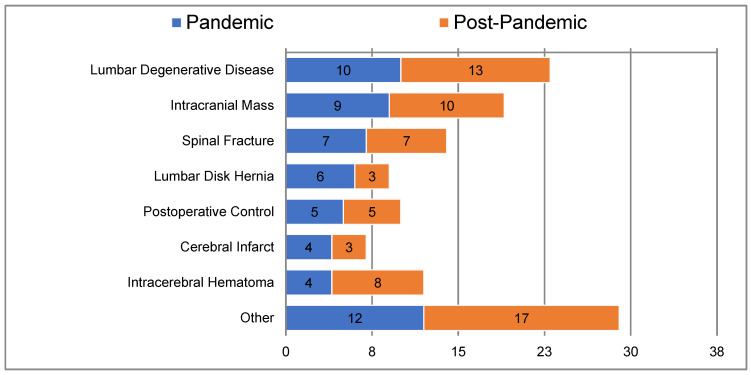
Diagnoses following consultation.

The most common diagnosis following consultation was lumbar degenerative disease, with occurrences of 17.5% and 19.7% in the pandemic and post-pandemic periods, respectively (Figure 2). During the pandemic, 26.3% of patients were recommended for physical therapy and rehabilitation, 24.6% were advised to undergo surgery, 19.3% received neurology consultations, and 17.5% received medical treatment.

In the post-pandemic period, 37.9% were recommended for surgery, 16.7% for neurology, 13.6% for physical therapy and rehabilitation, and 7.6% for medical treatment (Table 4). Overall, 56.4% of patients accepted surgery. Most patients who denied surgery were from oncology and neurology units in both periods. Among those who accepted surgery during the pandemic, 66% had spinal fractures, whereas in the post-pandemic period, normal pressure hydrocephalus and cranial masses were the most common conditions. The mean follow-up period was 33.2 months for the pandemic group and 13.8 months for the post-pandemic group. Postoperative mortality rates were 11% during the pandemic and 15.3% post-pandemic. Of the patients hospitalized for surgery, 71% were admitted to the orthopedic clinic, and new motor deficits were present in 85% of patients recommended for surgery. Of the 34 patients with a history of surgery, 44% had undergone intracranial mass surgery, while 38% had lumbar surgery. No inappropriate consultations were observed during the pandemic period, except for one patient consulted for empyema in the frontal sinus. Overall mortality rates were recorded as 40.4% during the pandemic and 30.3% post-pandemic.

## 4. Discussion

Current findings indicate a reduction in neurosurgery outpatient consultations from the emergency department during the COVID-19 pandemic. Meyer et al., in their study conducted in Southern Arizona, New Mexico, Texas, and California at a 649-bed tertiary-level, level I trauma center serving a referral population of 2 million, categorized neurosurgical consultations into “pre-pandemic” and “pandemic” periods, with ED patients either walking in or being transferred in. They demonstrated a significant reduction in the number of consultations during the pandemic period, with a 16% decrease compared to pre-pandemic levels. Furthermore, they reported a 10% decrease in ED consultations for trauma during the COVID-19 crisis. Overall, a 33% reduction in total ED consultations across all medical and surgical specialties was observed during the pandemic [14]. Similarly to our study, they defined the pandemic period as spanning from March 2020 to March 2022. In a retrospective study conducted at a level I trauma center in Miami, a 20% reduction in total neurosurgical consultations was observed in March 2020 compared to the pre-pandemic months of March and April 2016–2019. After the statewide “stay-at-home” order was issued in April 2020, they reported a significant 62% decrease in neurosurgical consultations for neurotrauma. Additionally, they demonstrated a significant 84% reduction in the number of surgeries performed for neurotrauma in April 2020 [15]. A study conducted across six neurosurgery departments in the Veneto region of Italy, analyzing surgical and inpatient procedures in March from 2016 to 2020, revealed a 25% reduction in cases of traumatic brain injury and a 35% reduction in spinal trauma at the onset of the pandemic. Furthermore, hospitalizations and surgical interventions for degenerative spinal conditions were reported to have decreased by approximately 50% [16]. Consistent with existing literature, we observed a decrease in outpatient neurosurgery services during this period, which prevented routine follow-ups for patients with prior surgeries. Notably, however, this study found a significant increase in consultations for patients with a history of surgery (*p* = 0.02). Additionally, the number of elective surgical procedures denied, as treatments for patients needing elective intervention were delayed [22]. Due to COVID-19’s severity and high transmission rates among geriatric patients, many avoided hospital visits for non-urgent issues, fearing exposure [24]. This study supports this observation, with the rate of consultations due to inadequate medical treatment recorded at approximately 23.4%. Evidence-based practice in spine diseases is often affected by psychosocial factors, such as patient and family attitudes, in addition to medical expertise [28,29]. Although a higher rate of inadequate medical treatment might be expected in geriatric populations due to their comorbidities, polypharmacy, and multidisciplinary care needs, the communication between departments in our hospital likely limited the percentage of patients requiring further intervention to 23.4%. While inappropriate neurosurgery consultations are reported at rates of 10-25% in the literature [30,31,32], in this study, only one consultation was identified as inappropriate. A study in Oman reported that one-third of geriatric neurosurgery patients had trauma-related conditions [1]. In the present study, trauma history was present in 15.4% of consulted patients, aligning with the literature when considering that emergency or direct neurosurgery admissions were excluded. Pandemic-period studies have reported a decrease in emergency trauma consultations [7,14,15]. However, some studies noted an increase in trauma cases, especially in patients over 80, in trauma center settings [17]. We observed a rise in trauma-related consultations post-pandemic, likely due to social isolation and lockdown effects during the pandemic. This period caused many patients to avoid seeking hospital care for conditions such as lumbar disc herniation and lumbar degenerative disease [18]. Koester et al. suggested that although emergency department visits decreased, the incidence of urgent neurosurgical or medical conditions, such as cerebral infarction, intracerebral hematoma, epilepsy, or acute hydrocephalus, likely remained constant. They proposed that the decrease in benign pathology visits could be attributed to patient fears of COVID-19 exposure [7]. Similarly, Meyer et al. reported a 20% decrease in degenerative cases during the pandemic, while consultations for subarachnoid hemorrhage and brain tumors remained stable [14]. Studies in different regions such as England, France, and Canada have shown comparable results [19,33,34]. In addition, a study of neurosurgery outpatient referrals found that most referrals were from neurology, physical therapy and rehabilitation, and orthopedic clinics [35]. Studies evaluating geriatric neurosurgery patients have identified traumatic brain injury, lumbar degenerative diseases, oncological conditions, and vascular pathologies as common conditions [1]. Another study found that about half of inpatient geriatric consultations involved internal medicine or related specialties [36]. Neurology was the most frequently requested consultation department in both the pandemic and post-pandemic periods, followed by internal medicine and its sub-specialties. This finding aligns with reported consultation patterns. While the increase in trauma cases was not statistically significant between periods, there was a notable increase in orthopedic consultations post-pandemic. Additionally, with elective surgeries delayed during the pandemic, consultations for preoperative evaluations for prior neurosurgical procedures rose significantly in the post-pandemic period (*p* = 0.042). Similarly, all patients undergoing surgery for normal pressure hydrocephalus were treated in the post-pandemic period. Consultations for back and leg pain significantly decreased post-pandemic, possibly due to the normalization of outpatient services, which allowed patients with lumbar degenerative diseases to visit neurosurgery clinics directly. This trend was also observed in treatment recommendations. Physical therapy and rehabilitation recommendations decreased from 26.3% during the pandemic to 13.6% post-pandemic, potentially due to restricted physical therapy and rehabilitation access during the pandemic and improved access afterward. A pandemic-era study suggested that postponed elective surgeries could lead to an increased post-pandemic neurosurgery workload, which our findings support [7]. While the rate of recommended surgeries increased post-pandemic, the frequency of performed surgical procedures remained unchanged. Geriatric trauma patients generally experience higher complication and mortality rates, adverse discharge outcomes, and longer hospital stays, with increased morbidity and mortality attributed to factors such as age-related physiological changes, comorbidities, polypharmacy, and frailty [1,22,37]. An evaluation of 1221 geriatric neurosurgical procedures at the Henry Ford Hospital found the highest mortality rate (41.3%) in intracerebral hematoma cases [38]. Another study reported a decrease in surgical mortality from 12% to 0.3% over 20 years, attributed to advances in surgical techniques, greater knowledge of geriatric medicine, and improved anesthesia methods [25]. A study evaluating geriatric surgeries reported mortality rates of 0.8% for elective and 10.1% for emergency procedures [26]. In this cohort, postoperative mortality was 13.6%, with no significant difference between periods. In a study examining the 18-month mortality of geriatric patients aged 75 and older hospitalized during the pandemic, mortality was 50.8% for COVID-19-positive patients and 66% for COVID-19-negative patients [39]. In our series, mortality rates were 40.4% during the pandemic and 30.3% afterward. Considering the average follow-up period, there was no significant difference in overall mortality between the two periods (*p* = 0.244). Neurosurgical procedures are often perceived as major interventions, not only by the public but also by healthcare professionals [27]. Similarly, a study evaluating geriatric patients recommended for major procedures, such as cardiac surgery, found a 40% surgery refusal rate [40]. In this study, 43.6% of geriatric patients recommended for surgery denied the procedure. It is frequently reported that family members refuse neurosurgical procedures for elderly patients due to concerns about their tolerance for such major interventions [27]. Sokas et al., in interviews with geriatric patients post-surgery, found that patients viewed surgery as a choice for extended life rather than as a risk of death [41]. Consequently, geriatric patients, who are often excluded from decision-making due to impaired mental status, may be denied neurosurgical procedures that could improve or extend their lives. As this study is a retrospective review from a single institution, potential observer and interpretation biases may have influenced our results, and these findings may not fully represent experiences across other hospitals. This study is limited by its focus on neurosurgery consultations among inpatients only.

## 5. Conclusions

We believe our analysis highlights important considerations in the field of geriatric neurosurgery. The current study demonstrates distinct shifts in geriatric neurosurgery consultation patterns post-pandemic. Although patient visits to the emergency department decreased during the pandemic due to various motivations, healthcare professionals maintained a consistent rate of neurosurgery consultation requests. This study found no reduction in neurosurgery consultations requested from inpatient departments during the pandemic. Future research that includes emergency, outpatient, inpatient, and intensive care consultations for neurosurgical care in geriatric patients could offer more comprehensive insights, supporting clinicians in optimizing referrals and enhancing the quality of neurosurgery services for this vulnerable population. Despite the high prevalence of comorbidities in geriatric patients, appropriate neurosurgical referrals significantly improve treatment success, enhance quality of life and mobility, and reduce mortality. We therefore recommend earlier and more attentive referrals to neurosurgery for elderly patients with relevant symptoms to facilitate timely and effective interventions.

## Figures and Tables

**Table 1:** Patient demographics and consultation patterns.

	Pandemic (n = 57)	Post-Pandemic (n = 66)	*p*-Value
Male	23 (40.4%)	36 (54.5%)	0.116
Age	73.45 ± 8.08 (65–93)	71.09 ± 5.22 (65–94)	0.416
COVID-19-Positive	15 (26.3%)	7 (10.6%)	0.023
Mortality (COVID-19-Positive)	11 (19.3%)	6 (9.1%)	0.102
Mortality (Total)	23 (40.4%)	20 (30.3%)	0.244
Hospitalization for Surgery	3 (5.3%)	14 (21.2%)	0.011
Surgery Recommendation	14 (24.6%)	25 (37.9%)	0.113
Denied Surgery	5 (8.8%)	12 (18.2%)	0.132
Surgical Intervention Performed	9 (15.8%)	13 (19.7%)	0.573
Surgical Mortality	1 (1.8%)	2 (3%)	1.000
New Motor Deficit	22 (38.6%)	29 (43.9%)	0.549
Inadequate Medical Treatment	8 (14%)	11 (16.7%)	0.687
Previous Surgery History	10 (17.5%)	24 (36.4%)	0.020
Trauma History	6 (10.5%)	13 (19.7%)	0.116

**Table 2:** Primary admission diagnoses at initial consultation.

Diagnosis	Pandemic (n = 57)	Post-Pandemic (n = 66)	*p*-Value
Malignancy	11 (19.3%)	16 (24.2%)	0.509
Hematologic Malignancy	7 (12.3%)	6 (9.1%)	0.566
Pneumonia	6 (10.5%)	4 (6.1%)	0.511
Other	5 (8.8%)	7 (10.6%)	0.732
Renal Failure	4 (7%)	5 (7.6%)	1.000
General Health Deterioration	4 (7%)	4 (6.1%)	1.000
Epilepsy	4 (7%)	4 (6.1%)	1.000
Headache	4 (7%)	2 (3%)	0.414
Multiple Myeloma	3 (5.3%)	2 (3%)	0.662
Diabetes Mellitus	3 (5.3%)	3 (4.5%)	1.000
Intracerebral Hematoma	3 (5.3%)	6 (9.1%)	0.502
Orthopedic Fracture	3 (5.3%)	7 (10.6%)	0.337

**Table 3:** Reasons for consultation.

Reason for Consultation	Pandemic (n = 57)	Post-Pandemic (n = 66)	*p*-Value
Back and Leg Pain	21 (36.8%)	13 (19.7%)	0.034
Motor Weakness	13 (22.8%)	17 (25.8%)	0.704
Headache	6 (10.5%)	8 (12.1%)	0.781
Mental Confusion	5 (8.8%)	4 (6.1%)	0.951
Other	5 (8.8%)	6 (9.1%)	0.732
Epilepsy	4 (7%)	6 (9.1%)	0.751
Neck and Arm Pain	3 (5.3%)	2 (3%)	0.662
Previous Surgery History	0	10 (15.2%)	0.002

**Table 4:** Recommended treatment modalities or departments referred.

Treatment/Department	Pandemic (n = 57)	Post-Pandemic (n = 66)	*p*-Value
Physical Therapy and Rehabilitation	15 (26.3%)	9 (13.6%)	0.077
Surgery	14 (24.6%)	25 (37.9%)	0.113
Neurology	11 (19.3%)	11 (16.7%)	0.704
Medical Treatment	10 (17.5%)	5 (7.6%)	0.092
Oncology	5 (8.8%)	6 (9.1%)	0.951
Other	2 (3.5%)	10 (15.2%)	0.030

## Data Availability

The original contributions presented in this study are included in the article. Further inquiries can be directed to the corresponding author.

## References

[1] Al-Saadi T., Al-Mirza A., Al-Taei O., Al-Saadi H. (2022). Geriatric neurosurgery in high-income developing countries: A Sultanate of Oman experience. Psychiatry Int..

[2] United Nations (2019). World Population Ageing. https://www.un.org/en/development/desa/population/publications/pdf/ageing/WorldPopulationAgeing2019-Report.pdf.

[3] Orimo H., Ito H., Suzuki T., Araki A., Hosoi T., Sawabe M. (2006). Reviewing the definition of “elderly”. Geriatr. Gerontol. Int..

[4] Schmidt E., Balardy L., Geeraerts T., Costa N., Bowers C.A., Hamilton M. (2020). Geriatric Neurosurgery: The Unfolding of A New Subspecialty. Neurosurg. Focus.

[5] Cloyd J.M., Acosta F.L., Ames C.P. (2008). Complications and outcomes of lumbar spine surgery in elderly people: A review of the literature. J. Am. Geriatr. Soc..

[6] Khu K.J.O., Chan K.I.P., Pascual J.S.G., Hernandez M.A.L.U. (2024). Neurosurgery in the elderly: Findings from a cohort in the Philippines. J. Clin. Neurosci..

[7] Koester S.W., Catapano J.S., Ma K.L., Kimata A.R., Abbatematteo J.M., Walker C.T., Cole T.S., Whiting A.C., Ponce F.A., Lawton M.T. (2021). COVID-19 and Neurosurgery Consultation Call Volume at a Single Large Tertiary Center With a Propensity-Adjusted Analysis. World Neurosurg..

[8] Axenhus M., Schedin-Weiss S., Tjernberg L., Winblad B. (2024). The impact of the COVID-19 pandemic on neurosurgery in the elderly population in Sweden. BMC Public Health.

[9] Hemingway J.F., Singh N., Starnes B.W. (2020). Emerging practice patterns in vascular surgery during the COVID-19 pandemic. J. Vasc. Surg..

[10] Jean W.C., Ironside N.T., Sack K.D., Felbaum D.R., Syed H.R. (2020). The impact of COVID-19 on neurosurgeons and the strategy for triaging non-emergent operations: A global neurosurgery study. Acta Neurochir..

[11] Liveris A., Stone M.E., Markel H., Agriantonis G., Bukur M., Melton S., Roudnitsky V., Chao E., Reddy S.H., Teperman S.H. (2022). When New York City was the COVID-19 pandemic epicenter: The impact on trauma care. J. Trauma Acute Care Surg..

[12] World Health Organization. https://covid19.who.int.

[13] Budak O., Erdal N., Filiz M. (2023). Measuring the anxiety level in the post-pandemic normalization process: The case of Türkiye. Turk. Res. J. Acad. Soc. Sci..

[14] Meyer B.M., de Andrada Pereira B., Mamaril-Davis J., Hurlbert R.J. (2023). Consultations during COVID: Effects of a pandemic on neurosurgical care. World Neurosurg..

[15] Figueroa J.M., Boddu J., Kader M., Berry K., Kumar V., Ayala V., Vanni S., Jagid J. (2021). The effects of lockdown during the severe acute respiratory syndrome coronavirus 2 (SARS-CoV-2) pandemic on neurotrauma-related hospital admissions. World Neurosurg..

[16] Raneri F., Rustemi O., Zambon G., Del Moro G., Magrini S., Ceccaroni Y., Basso E., Volpin F., Cappelletti M., Lardani J. (2020). Neurosurgery in times of a pandemic: A survey of neurosurgical services during the COVID-19 outbreak in the Veneto region in Italy. Neurosurg. Focus.

[17] Agyemang K., Rose A., Baig S., Al Salloum L., Osman A.A., Steckler F., Barrett C. (2021). Neurosurgery in octogenarians during the COVID-19 pandemic: Results from a tertiary care trauma centre. Interdiscip. Neurosurg..

[18] Lima D.L., Pereira X., Dos Santos D.C., Camacho D., Malcher F. (2020). Where are the hernias? A paradoxical decrease in emergency hernia surgery during COVID-19 pandemic. Hernia.

[19] Kansagra A.P., Goyal M.S., Hamilton S., Albers G.W. (2020). Collateral effect of COVID-19 on stroke evaluation in the United States. N. Engl. J. Med..

[20] Proust F., Gérardin E., Derrey S., Lesvèque S., Ramos S., Langlois O., Tollard E., Bénichou J., Chassagne P., Clavier E. (2009). Interdisciplinary treatment of ruptured cerebral aneurysms in elderly patients. J. Neurosurg..

[21] Sacko O., Sesay M., Roux F.E., Riem T., Grenier B., Liguoro D., Loiseau H. (2007). Intracranial meningioma surgery in the ninth decade of life. Neurosurgery.

[22] Eagles D., Godwin B., Cheng W., Moors J., Figueira S., Khoury L., Fournier K., Lampron J. (2020). A systematic review and meta-analysis evaluating geriatric consultation on older trauma patients. J. Trauma Acute Care Surg..

[23] Esmaeili E.D., Azizi H., Sarbazi E., Khodamoradi F. (2023). The global case fatality rate due to COVID-19 in hospitalized elderly patients by sex, year, gross domestic product, and continent: A systematic review, meta-analysis, and meta-regression. New Microbes New Infect..

[24] Ulugerger Avci G., Bektan Kanat B., Suzan V., Can G., Korkmazer B., Karaali R., Tabak F., Borekci S., Aygun G., Yavuzer H. (2022). Clinical outcomes of geriatric patients with COVID-19: Review of one-year data. Aging Clin. Exp. Res..

[25] Chibbaro S., Di Rocco F., Makiese O., Mirone G., Marsella M., Lukaszewicz A.C., Vicaut E., Turner B., Hamdi S., Spiriev T. (2011). Neurosurgery and elderly: Analysis through the years. Neurosurg. Rev..

[26] Bligh E.R., Sinha P., Smith D., Al-Tamimi Y.Z. (2020). Thirty-day mortality and survival in elderly patients undergoing neurosurgery. World Neurosurg..

[27] Okunlola A.I., Okunlola C.K., Babalola O.F., Orewole T.O., Akinmade A., Kofoworola O.O. (2021). Geriatric neurosurgery in a suburban community: A preliminary review of a single neurosurgeon experience. Interdiscip. Neurosurg..

[28] Cherkin D.C., Deyo R.A., Street J.H., Barlow W. (1996). Predicting poor outcomes for back pain seen in primary care using patients’ own criteria. Spine.

[29] Buchbinder R., Staples M., Jolley D. (2009). Doctors with a special interest in back pain have poorer knowledge about how to treat back pain. Spine.

[30] Debono B., Sabatier P., Koudsie A., Buffenoir K., Hamel O. (2017). Managing spine surgery referrals: The consultation of neurosurgery and its nuances. Neurochirurgie.

[31] Lichtenstein H., Winograd C.H. (1984). Geriatric consultation: A functional approach. J. Am. Geriatr. Soc..

[32] Van Essen T.A., Heeringa J.J., Muizelaar J.P. (2010). (In)appropriate neurosurgical consultation. Clin. Neurol. Neurosurg..

[33] Bernat A.L., Giammattei L., Abbritti R., Froelich S. (2020). Impact of COVID-19 pandemic on subarachnoid hemorrhage. J. Neurosurg. Sci..

[34] Diestro J.D.B., Li Y.M., Parra-Fariñas C., Sarma D., Bharatha A., Marotta T.R., Spears J. (2020). Letter to the editor: Aneurysmal subarachnoid hemorrhage: Collateral damage of COVID?. World Neurosurg..

[35] Ülkü G., Karaavcı N.Ç., Elbir Ç., Demirtaş O.K. (2024). Retrospective evaluation of neurosurgery outpatient services in 3 different tertiary care hospitals in Turkey. World Neurosurg..

[36] Matsushima K., Schaefer E.W., Won E.J., Armen S.B., Indeck M.C., Soybel D.I. (2014). Positive and negative volume-outcome relationships in the geriatric trauma population. JAMA Surg..

[37] Bryant E.A., Tulebaev S., Castillo-Angeles M., Moberg E., Senglaub S.S., O’mara L., McDonald M., Salim A., Cooper Z. (2019). Frailty identification and care pathway: An interdisciplinary approach to care for older trauma patients. J. Am. Coll. Surg..

[38] Dujovny M., Charbel F., Kim Berman S., Diaz F.G., Malik G., Ausman J.I. (1987). Geriatric neurosurgery. Surg. Neurol..

[39] Claes M., Genet B., Rouet A., Boutitie L., Parramore P., Hardy É., Thomas C., Zerah L., Vallet H. (2024). Mortality and functional outcomes 18 months after hospitalization for COVID-19 in geriatric patients: A multicentric cohort study. BMC Geriatr..

[40] Iung B., Cachier A., Baron G., Messika-Zeitoun D., Delahaye F., Tornos P., Gohlke-Ba C., Boersma E., Ravaud P., Vahanian A. (2005). Decision-making in elderly patients with severe aortic stenosis: Why are so many denied surgery?. Eur. Heart J..

[41] Sokas C., Yeh I.M., Coogan K., Bernacki R., Mitchell S., Bader A., Ladin K., Palmer J.A., Tulsky J.A., Cooper Z. (2021). Older adult perspectives on medical decision making and emergency general surgery: “It had to be done”. J. Pain Symptom Manag..

